# A Calibration-Free Method Based on Grey Relational Analysis for Heterogeneous Smartphones in Fingerprint-Based Indoor Positioning

**DOI:** 10.3390/s19183885

**Published:** 2019-09-09

**Authors:** Shuai Zhang, Jiming Guo, Nianxue Luo, Di Zhang, Wei Wang, Lei Wang

**Affiliations:** 1 School of Geodesy and Geomatics, Wuhan University, Wuhan 430079, China; 2016102140015@whu.edu.cn (S.Z.); dzhang@sgg.whu.edu.cn (D.Z.); wangw_whu@163.com (W.W.); 2 Research Center for High Accuracy Location Awareness, Wuhan University, Wuhan 430079, China; 3 State Key Laboratory of Information Engineering in Surveying, Mapping and Remote Sensing, Wuhan University, Wuhan 430079, China; lei.wang@whu.edu.cn

**Keywords:** Wi-Fi fingerprint, grey relational analysis, heterogeneous smartphones, indoor positioning

## Abstract

The fingerprint method has been widely adopted in Wi-Fi indoor positioning because of its advantage in non-line-of-sight channels between access points (APs) and mobile users. However, the received signal strength (RSS) during the fingerprint positioning process generally varies due to the dissimilar hardware configurations of heterogeneous smartphones. This difference may degrade the accuracy of fingerprint matching between fingerprint and test data. Thus, this paper puts forward a fingerprint method based on grey relational analysis (GRA) to approach the challenge of heterogeneous smartphones and to improve positioning accuracy. Initially, the grey relational coefficient (GRC) between the RSS comparability sequence of each reference point (RP) and the RSS reference sequence of the test point (TP) is calculated. Subsequently, the grey relational degree (GRD) between each RP and TP is determined on the basis of GRC, and the K most relational RPs are selected in accordance with the value of GRD. Finally, the user location is determined by weighting the K most relational RPs that correspond to the coordinates. The main advantage of this GRA method is that it does not require device calibration when handling heterogeneous smartphone problems. We further carry out extensive experiments using heterogeneous Android smartphones in an office environment to verify the positioning performance of the proposed method. Experimental results indicate that the proposed method outperforms the existing ones no matter whether heterogeneous smartphones are used.

## 1. Introduction

At present, location-based service requirements have rapidly increased. Nowadays, the global navigation satellite system (GNSS) can satisfactorily addressed location service requirements in outdoor environments. However, GNSS cannot meet the indoor positioning requirement because of the fading signal and multipath effect in indoor environments [1,2,3]. Several technologies, such as ultrasound, Wi-Fi, RFID, Bluetooth, ZigBee, geomagnetic positioning, and ultrawide band, have been used for indoor positioning. Amongst these technologies, indoor positioning with Wi-Fi has attracted considerable attention because it does not require additional equipment and has low cost [4]. Several methods, such as angle of arrival (AOA), time of arrival (TOA), time difference of arrival (TDOA), and fingerprint method, have been adopted in Wi-Fi indoor positioning [5,6,7,8,9,10,11]. AOA, TOA, and TDOA require point-to-point distance or angle information. These methods have simple calculations, but they are developed under the condition of line-of-sight (LOS) channels between access points (APs) and mobile users. The fingerprint method does not require LOS propagation between APs and mobile users and has been widely adopted for indoor positioning [12,13,14,15,16,17,18,19,20,21].

Smartphones are becoming highly intelligent with the development of science and technology. Smartphones can help in solving many problems, such as navigation and positioning, which have become a part of people’s lives. The use of a smartphone-based positioning system is convenient because of the popularity and abundance of various embedded sensors in smartphones. On this basis, several studies investigated smartphone-based indoor positioning and various solutions have been presented in the literature [22,23,24]. Common fingerprint methods firstly collect the received signal strength (RSS) data and corresponding coordinate information as the fingerprint database at each reference point (RP) in the offline phase. In the online phase, the user’s location can be determined with the best-fitted fingerprint by comparing the online-measured RSS with the fingerprint database [25,26,27,28,29]. Several conventional matching algorithms, such as K nearest neighbour (KNN) [30], weighted KNN (WKNN) [31], and Bayesian probability algorithm [32], can be used as fingerprint methods. Meanwhile, Euclidean distance is widely adopted in KNN and WKNN. RSS data are usually collected using the same smartphone in the offline and online phases when these conventional algorithms are used for fingerprint matching; otherwise, heterogeneous smartphone problems can be hardly avoided and positioning accuracy is degraded. Such phenomenon is caused by the collected RSS data, which are influenced by certain hardware factors, such as antenna gains, antenna location, and different WLAN chipsets [33]. The heterogeneous smartphone problem mentioned in this work indicates that the RSS data are collected by using heterogeneous smartphones in the offline and online phases.

Haeberlen et al. [34] and Kjærgaard et al. [35] presented different device calibration methods, namely, manual, quasi-automatic, and automatic, to solve this problem. Such device calibration methods are used to mitigate the influence of RSS difference due to the use of heterogeneous smartphones. These methods are time consuming and have low scalability as the number of new smartphones increases. They acquire the calibration parameters by training samples. Laoudias et al. [36] presented a self-calibration method, which did not need to collect a series of RSS data at several known locations with a pair of heterogeneous devices and which did not require any user intervention for calibration. The online RSS data of user device were calibrated and updated in the positioning process. Although it is a self-calibration method (SC), the method still needs a calibration process, which adds the complexity of positioning. Moreover, the accuracy is not adequate at the beginning of positioning because the device has not been calibrated at the beginning. Tsui et al. [37] reduced the training time of smartphones to address the time-consuming problem for realising rapid device calibration. Nevertheless, it is still impractical for all kinds of smartphones to be trained. Therefore, device calibration-free methods were investigated to reduce the effects caused by the use of heterogeneous smartphones.

A type of device calibration-free method, which does not need to collect training data for device calibration, utilises the RSS difference between APs or locations to mitigate the effect of using heterogeneous smartphones. Shu et al. [38] proposed a gradient fingerprinting method, which leveraged RSS differences amongst locations. Firstly, the method creates a gradient-based fingerprint map by comparing the absolute RSS values at nearby positions. Secondly, it runs an online extended particle filter to determine the user position. Dong et al. [39] used the signal strength difference between pairwise APs, which are called difference of signal strength (DIFF), to reduce the impact of using heterogeneous smartphones. Hossain et al. [40] proposed an enhanced method called signal strength difference (SSD), which selected a DIFF independent subset to reduce computational overhead. Liu et al. [41] and Kjærgaard et al. [42] utilised signal strength ratios, such as hyperbolic location fingerprinting (HLF), as fingerprints to overcome the hardware variance problem and to minimise the positioning error. The device calibration-free methods based on RSS difference reduce time consumption and increase adaptability for heterogeneous smartphones. However, these methods are used at the expense of losing certain discriminative information. Fang et al. [43] established a novel positioning feature called delta-fused principal strength to improve the accuracy and to solve the problem of Wi-Fi positioning when heterogeneous smartphones were used. The main idea utilises the complementary advantages of various positioning features. However, the aforementioned methods based on the RSS difference led to a potential reduction in accuracy when RSS data in the offline and online phases are collected using homogenous smartphones [44].

Another type of device calibration-free method uses the similarity between fingerprint and online RSS data instead of the conventional Euclidean distance. Han et al. [45] and Caso et al. [46] presented a fingerprint algorithm based on cosine similarity and Pearson’s correlation, respectively. These approaches do not require device calibration and complicated computation. However, maintaining a desired positioning accuracy for these methods in the real-world environment is difficult because the methods assume that the RSS differences caused by heterogeneous mobile devices are constants. In complex indoor environments, the positioning effect of these methods are not ideal.

In addition, other device calibration-free methods were presented. Yang et al. [47] built RSS datasets from all APs at test point (TP) and each RP, and the subset of each AP in each dataset contained the APs with lower RSSs than it. The similarity of TP and RP fingerprints can be determined by the accumulating number of common subset sizes of each AP in the dataset for positioning. The method uses only relative relationship information among RSS values rather than absolute RSS values. Chen et al. [48] proposed an algorithm based on the idea of longest common subsequences (LCS) to deal with the AP changes and RSS variation of heterogeneous devices in an AP-intensive environment. The method utilizes similarities between TP and RP fingerprints for positioning. The similarity of two fingerprints can be determined by the length of the longest common subsequence between TP and RP fingerprint sequences ordered by signal strengths. However, the two methods need a lot of APs deployed, and the positioning performance is not ideal when the number of APs is not enough.

Since the grey system theory was proposed in 1982, it has been widely applied in social and economic fields because of its advantages in evaluating complex systems with multiple criteria and multiple factors, such as investment returns of various sectors of national economy, analysis of regional economic advantages, and adjustment of industrial structure [49,50]. Grey system theory puts forward the concept of GRA for a system. GRA is a multifactor analysis method. The basic principle is to determine the close degree of multifactor in a system by comparing the geometric relationship between reference and comparability sequences. The closer the geometric shape of sequence curves is, the larger the GRD between them is. Conversely, if the curves are remarkably different, the GRD between them becomes small. Thus, the GRA is also used by researchers in positioning and location recognition. Du et al. [51] investigated a novel TOA-based location estimation algorithm by using cellular geometric analysis and GRA. Xiao et al. [52] realised human location recognition based on GRA. This method could determine whether the user’s location is an indoor or outdoor environment in order to decide whether to offer wireless network access. The grey systems are called “grey”, implying poor, incomplete, and uncertain information. Therefore, GRA is suitable for small data samples; nonlinear, existing uncertain information; and samples without typical distribution regulation. When the fingerprint method is used for positioning in heterogeneous smartphones, the RSS sample only contains RSS data from several APs at each RP in the fingerprint database and there is not enough RSS data for the sample. The RSS samples at each TP and RP do not have typical distribution regulation. The pair curves consisting of RSS data from all APs using heterogeneous smartphones at the same location are not absolutely similar, and a nonlinear relation between them in the real-world environment exists. In the positioning process, the relational degree between each RP and the TP is also uncertain. In these cases, GRA is beneficial for solving the heterogeneous problem of smartphones in fingerprint methods. Therefore, a device calibration-free fingerprint method based on GRA is proposed.

In this method, firstly, average RSS data received from all APs at the TP form a reference sequence and average RSS data received from all APs at each RP form each comparability sequence. Then, the grey relational coefficient (GRC) between the RSS comparability and reference sequence is calculated by the RSS difference between curves, which is used to describe the correlation between them. Next, GRD is calculated by the GRC between reference and comparability sequences. The closer the comparability sequence curve is to the reference sequence curve, the larger the GRD between them is. Subsequently, the K most relational RPs are selected. Finally, the user location is determined by weighting K most relational RPs that correspond to the coordinates. This above-proposed method guarantees the completeness of RSS information. Moreover, the method is applicable to the nonlinear relationship between vectors and has no requirement for typical distribution regulation of vectors. This method avoids the fingerprint matching errors of conventional positioning methods by using similarity of comparability and reference sequences. The proposed method can adapt to the changes of RSS data between two vectors better than other fingerprint methods in terms of similarity because the GRD is determined by the RSS difference between curves, which does not require complete similarity between curves. Another advantage of the method is that it can avoid missing the raw RSS fingerprint information compared with other RSS difference-based fingerprint methods. In addition, the proposed GRA method also has the advantages of less calculation and of a simple principle. The positioning performance of the proposed method is improved whether heterogeneous smartphones are used.

The remainder of this work is organised as follows: Section 2 analyses the RSS difference and positioning performance of conventional fingerprint methods when heterogeneous smartphones are used. Section 3 illustrates the proposed GRA-based device calibration-free fingerprint method in detail. Section 4 evaluates the experimental results of the GRA-based fingerprint method. Section 5 summarises our conclusions.

## 2. Impact of Heterogeneous Smartphones on Fingerprint Positioning

In this section, the RSS difference of heterogeneous smartphones is analysed and the influence of heterogeneous smartphones on positioning accuracy is discussed. The result indicates that the received RSS data have large differences and that the positioning performance of conventional fingerprint algorithms is degraded when heterogeneous smartphones are used.

### 2.1. Difference Analysis of RSS Collected by Heterogeneous Smartphones

**Figure 1:**
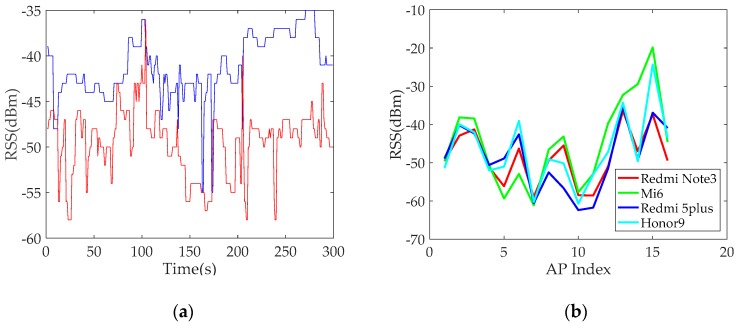
Variations in received signal strength (RSS) values resulting from different conditions: (**a**) The RSS value collection from the same access point (AP) using two heterogeneous smartphones at the same location and (**b**) the RSS value collection from the 16 APs using heterogeneous smartphones at the same location.

An experiment is carried out in a conference room to analyse the influence of heterogeneous smartphones on RSS. Two heterogeneous smartphones are used to measure the RSS data from the same AP and location. A total of 300 RSS data are collected within 5 min at a frequency of 1 Hz. Figure 1a shows the results. Differences in the RSS values are observed when heterogeneous smartphones are used, and the average difference of RSS data collected by two heterogeneous smartphones over a period of time is approximately 8 dBm.

Another experiment is designed to further analyse the difference of the collected RSS data from various APs in the same location using heterogeneous smartphones. Four heterogeneous smartphones are used to collect RSS data from 16 APs at the same location. RSS data are collected for 5 min at a frequency of 1 Hz. Figure 1b shows the results. Each curve consists of the average RSS data collected from 16 APs. The result also shows that the RSS data collected by using heterogeneous smartphones significantly vary, thereby further verifying the effect of smartphone heterogeneity on the RSS value. Figure 1b also shows that the measured RSS data from some APs using Mi 6 smartphones are the strongest but that the measured RSS data from other APs using Mi 6 smartphones are the weakest. The reason is that the collected RSS data from some APs using Mi 6 smartphones are incomplete and missing. We default the missing data for a constant of −95 dBm as preprocessing of the missing data. If part of the collected RSS data is missing, the average value from some APs over a period of time will be reduced. This figure clearly indicates that the curve shapes display a relatively consistent fluctuating trend even though differences between any pair of curves from various smartphones exist. However, any pair of curves from heterogeneous smartphones is not absolutely similar. Specifically, one curve cannot be absolutely coincident with another one via translation operations. The influence of RSS differences on positioning performance will be discussed in the next sections.

### 2.2. Influence of Added Test Constant on the Positioning Performance for the Same Smartphone

**Figure 2:**
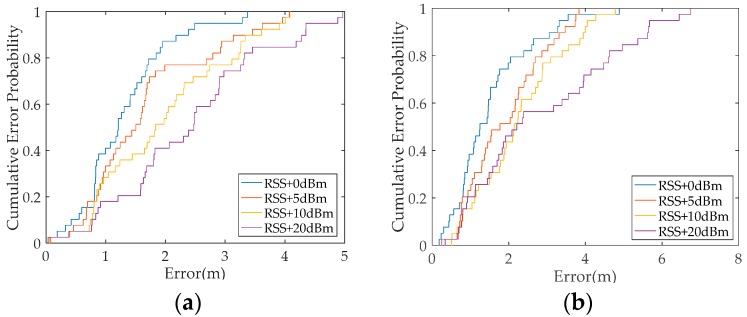
Positioning performance of conventional fingerprint methods when adding the test constant: (**a**) weighted K nearest neighbour (WKNN) algorithm and (**b**) Bayesian algorithm.

In the previous experiment, the RSS value has differences when heterogeneous smartphones are used. Thus, we hypothesize that differences between the received RSS in the offline and online phases affect positioning performance. An experiment is designed to verify this conjecture. The experiment is conducted in an office building of the School of Geodesy and Geomatics, Wuhan University. The office area measures 6.4 m × 12.8 m. Nine APs are distributed in the office as transmitters. A total of 28 RPs and 39 TPs are present in the office. RSS data are collected for 1 min at a frequency of 1 Hz at each RP and TP. RSS data are collected by using the same smartphone in the offline and online phases. Conventional algorithms are adopted in the experiment. The positioning performance is tested by adding several constants in the online phase on the basis of RSS data collected from TPs. Figure 2a shows the positioning results of all TPs. When the RSS data collected from TPs are truth values in the offline and online phases, the positioning accuracy of the WKNN algorithm is optimal. The positioning accuracy of WKNN algorithm is decreased when the RSS values from TPs add a constant of five compared with the truth values in the online phase. The positioning accuracy gradually decreases with the increase in the constant value (Figure 2a). The positioning results of the all TPs are calculated by using the Bayesian algorithm (Figure 2b). The results also show that the positioning accuracy of the Bayesian algorithm gradually decreases with the increase of the constant in the online phase. Therefore, the difference between the received RSS in the offline and online phases evidently affects the positioning performance.

### 2.3. Influence of Heterogeneous Smartphones in Conventional Positioning Methods

**Figure 3:**
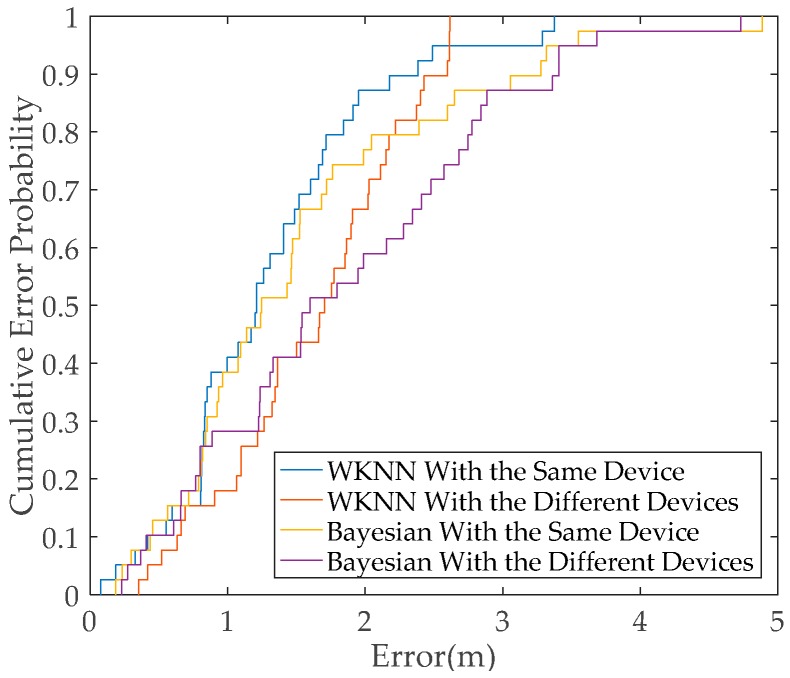
Comparison of the positioning performance of conventional methods in the same and different smartphones.

A new experiment is designed to further verify the influence of heterogeneous smartphones on positioning accuracy. The experimental setting is the same as that in Section 2.2. The RSS data are collected by using two heterogeneous smartphones in the offline and online phases. The WKNN and Bayesian algorithms are used for positioning in this experiment. In Figure 3, the test results show that the positioning performance of the WKNN algorithm using the same smartphone outperforms the WKNN algorithm when heterogeneous smartphones are used. The positioning performance of the Bayesian algorithm using the same smartphone also outperforms the Bayesian algorithm when heterogeneous ones are used. Accordingly, the positioning performance of conventional methods is not ideal and presents degradation when heterogeneous smartphones are used in the offline and online phases.

The RSS differences and positioning performances of conventional methods in heterogeneous smartphones are analysed. The results show that there is a difference between the RSS values and that the positioning performance of conventional methods are degraded when heterogeneous smartphones are used. Therefore, we aim to explore a novel fingerprint method to improve the positioning accuracy when heterogeneous smartphones are used. Accordingly, a GRA-based device calibration-free fingerprint method is proposed. The positioning performance of the proposed method will be analysed and compared in detail in the subsequent sections.

## 3. GRA-Based Fingerprint Method

GRA is a multifactor analysis method in grey system theory, which analyses uncertain relations between one main factor and all the other factors. GRA usually is used to determinate the relational degree according to the similarity of the geometric shape among factors. In the GRA-based fingerprint method, the GRD is used to determine user location through the similarity between the reference sequence and the comparability sequence. The closer the comparability sequence curve is to the reference sequence curve, the larger the GRD between them is.

A GRA-based device calibration-free fingerprint method is proposed to mitigate the impact of heterogeneous smartphones on positioning performance. The positioning performance is improved by the proposed method when heterogeneous smartphones are used. In this section, the GRA-based device calibration-free fingerprint method is described with an overall positioning flow.

### 3.1. Overview of GRA-Based Fingerprint Method

**Figure 4:**
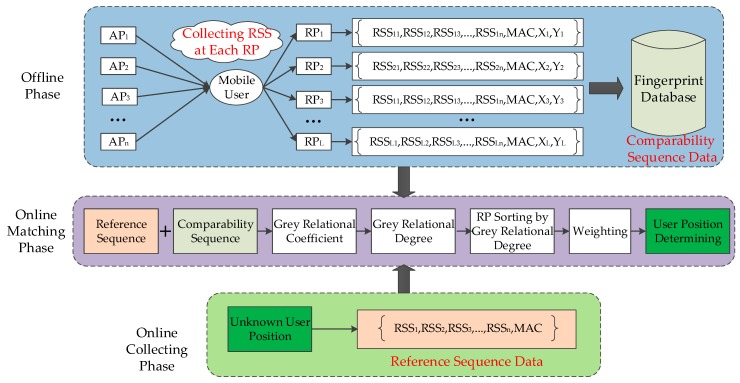
Overview of the fingerprint positioning method based on grey relational analysis.

**Figure 5:**
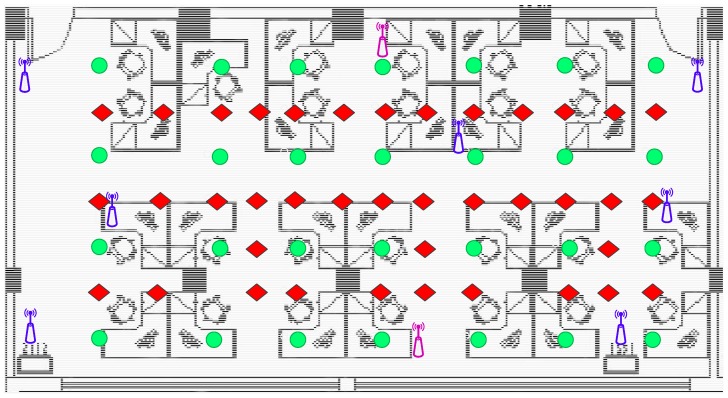
Experiment settings for indoor positioning: The green, circular symbols represent reference points (RPs), and the red, diamond symbols represent test points (TPs).

Fingerprint positioning is a widely used method for Wi-Fi indoor positioning because it does not require LOS conditions. Conventional fingerprint methods consist of two phases, namely, offline and online phases. In the offline phase, the location area is divided into grids. The RPs are deployed in the grids. The RSS values at each RP are collected at preset time intervals. The collected RSS data are used as fingerprint data in the offline phase. The RSS data, MAC addresses of Aps, and location information of RPs are used to develop the fingerprint database. In the online phase, the online RSS data measured at unknown user locations are firstly collected to determine user location. Secondly, the online RSS data and fingerprint of the fingerprint database are matched according to the conventional positioning algorithms, such as WKNN and Bayesian, to find the nearby RPs. Finally, the user location is estimated according to the corresponding coordinates of the selected RPs. In this work, the GRA-based method is proposed on the basis of fingerprint positioning. Figure 4 shows the processing procedure of the proposed method. Such a procedure can be divided into three parts, namely, offline, online collecting, and online matching phases. In the offline phase, the process of creating the fingerprint is the same as that of the conventional methods. In the online collecting phase, we only collect the RSS data at the TP. In the online matching phase, online RSS data are firstly used as a reference sequence and the fingerprint database is used as comparability sequences. The GRC between the RSS comparability sequence of RP and the RSS reference sequence of the TP is subsequently calculated, and the GRD is obtained by the GRC. Finally, the K most relational RPs are selected by using the GRD between each RP and TP. The user location is determined by weighting K coordinates associated with the K most relational RPs. Figure 5 illustrates the GRA-based fingerprint positioning method.

### 3.2. Implementation of Fingerprint Method Based on GRA

In this work, our approach consists of three phases, namely, offline, online collecting, and online matching phases. Firstly, a fingerprint database is developed by using the RSS data collected at each RP. Secondly, the online RSS data are collected at a TP. Finally, GRC and GRD between reference and comparability sequences are calculated and the user location is determined. Each phase is explained in the following sections.

The RSS data are collected from different APs at each RP to develop a fingerprint database. Each RP location is known. A total of 60 RSS data are collected within 1 min at each RP, and the average RSS value of each RP is calculated as fingerprint data. The fingerprint data, MAC addresses of all Aps, and location information of RPs are used to develop the fingerprint database. In Equation (1), $FD_{RP}$ is a matrix of the fingerprint data and $RSS_{i,j}$ is the average of the collected RSS data of the *j*th AP at the *i*th RP. *n* and *L* represent the total amount of deployed APs and RPs in the environment, respectively.

(1) $$FD_{RP}=\left[\begin{array}{ccccc} RSS_{1,1} & RSS_{1,2} & RSS_{1,3} & \cdots & RSS_{1,n}\\ RSS_{2,1} & RSS_{2,2} & RSS_{2,3} & \cdots & RSS_{2,n}\\ RSS_{3,1} & RSS_{3,2} & RSS_{3,3} & \cdots & RSS_{3,n}\\ \vdots & \vdots & \vdots & \cdots & \vdots \\ RSS_{L,1} & RSS_{L,2} & RSS_{L,3} & \cdots & RSS_{L,n} \end{array}\right]$$

In the fingerprint data, the RSS data vector from all APs at each RP is defined as a comparability sequence. The comparability sequences of all RPs form a comparability sequence matrix. In Equation (2), $CS_{RP}$ is a comparability sequence matrix and each column of the matrix represents a comparability sequence.

(2) $$CS_{RP}=F{D_{RP}}^{T}$$

The collected average RSS data from all APs at a TP is used as a reference sequence. As shown in Equation (3), $RS_{TP}$ is a reference sequence vector.

(3) $$RS_{TP}={\left(\begin{array}{ccccc} RSS_{1} & RSS_{2} & RSS_{3} & \cdots & RSS_{n} \end{array}\right)}^{T}$$

In grey relational analysis, reference sequence and comparability sequence cannot be compared directly since they may have different dimensions and magnitudes. Therefore, normalization is needed to translate the original sequence to a comparable sequence, which is generally dimensionless. Data normalization is also necessary when the sequence scatter range is large. According to the characteristics of data sequence, there are some normalized methods for grey relational analysis such as zero-mean normalization, min-max normalization, initialization method, and mean value method. In this paper, the units of data between original reference and comparability sequences are the same. Thus, the original sequences only need to be simply normalized by the most basic methodology to reduce the sequence scatter range, i.e., let the values of original sequence be divided by the mean of the sequence. In Equation (4), $RSS_{i,j}^{*}$ is a normalized result of the RSS data of the *j*th AP at the *i*th RP (*i* = 1, 2, …, *L*; *j* = 1, 2, …, *n*).

(4) $$RSS_{i,j}^{*}=\frac{RSS_{i,j}}{\frac{1}{n}\sum_{k=1}^{n}RSS_{i,k}}$$

In Equation (5), $RSS_{j}^{*}$ is a normalized processing result of the RSS data of the *i*th AP at a TP (*j* = 1, 2, …, *n*).

(5) $$RSS_{j}^{*}=\frac{RSS_{j}}{\frac{1}{n}\sum_{k=1}^{n}RSS_{k}}$$

From the geometry analysis, the GRD is the similarity between the reference and the comparability sequence curves. The closer the comparability sequence curve is to the reference sequence curve, the larger the GRD between them is. Conversely, if the curves are remarkably different, the GRD between them becomes small. Therefore, the RSS difference between curves can be used as a scale standard of GRD. Firstly, the RSS absolute differences between the elements of the comparability sequence of each RP and the elements of the reference sequence of TP are calculated in Equation (6) to determine the GRC. The maximum and minimum RSS differences are then obtained in all RSS differences between comparability and reference sequences (Equations (7) and (8)). Finally, GRC is calculated by using Equation (9).

(6) $$\Delta_{i,j}=|RSS_{j}^{*}-RSS_{i,j}^{*}|$$

where $\Delta_{i,j}$ is the RSS absolute difference between the *j*th AP at the *i*th RP (*i* = 1, 2, …, *L*; *j* = 1, 2, …, *n*) and the *j*th AP at the TP.

(7) $$\Delta_{min}=\overset{L}{\underset{i=1}{\min}}\overset{n}{\underset{j=1}{\min}}\Delta_{i,j}$$

(8) $$\Delta_{max}=\overset{L}{\underset{i=1}{\max}}\overset{n}{\underset{j=1}{\max}}\Delta_{i,j}$$

(9) $$r_{i,j}=\frac{\Delta_{min}+\rho \Delta_{max}}{\Delta_{i,j}+\rho \Delta_{max}}$$

where $r_{i,j}$ indicates the GRC between the RSS data of the *j*th AP at the *i*th RP (*i* = 1, 2, …, *L*; *j* = 1, 2, …, *n*) and RSS data of the *j*th AP at the TP. $\Delta_{max}$ is the maximum RSS difference in all RSS differences between the comparability and reference sequences, and $\Delta_{min}$ is the minimum one. ρ is the identification coefficient, which is a value between 0 and 1, and 0.5 is generally used. This identification coefficient is introduced artificially to improve the difference between GRCs.

The GRD represents the level of correlation between the reference sequence and the comparability sequence. Due to the GRD between each comparability sequence and reference sequence being reflected by *n* GRCs, GRD is not easy to compare when the correlation information is scattered. Therefore, it is necessary to centralize the correlation information. Average value is a method of information centralization. That is, the GRD between the comparability and reference sequences can be quantitatively reflected by the average value of the all GRCs of each factor between the comparability sequence and the reference sequence. GRD is calculated by the GRC between reference and comparability sequences.

(10) $$R_{i}=\frac{1}{n}\sum_{j=1}^{n}r_{i,j}$$

where $R_{i}$ is the GRD between the *i*th RP and TP (*i* = 1, 2, …, *L*). The value of $R_{i}$ represents the correlation level. If $R_{2}$ is larger than $R_{1}$, then the correlation between the second RP and TP is higher than that between the first RP and TP.

We sort amongst RPs, select the K most relational RPs by GRD between each RP and TP, and calculate the weight of the K RP with the corresponding GRD in Equation (11) to obtain the user location. In Equation (12), the user location is obtained by weighting K coordinates associated with the K most relational RPs.

(11) $$w_{i}=\frac{R_{i}}{\sum_{t=1}^{K}R_{t}}$$

where $R_{i}$ is the GRD between the *i*th RP and the TP and $w_{i}$ represents the weight of the *i*th RP in the K RPs (*i* = 1, 2, …, *K*).

(12) $$(x,y)=\sum_{i}^{K}w_{i}(x_{i},y_{i})$$

where (x,y) denotes the coordinates of the user location and $\left(x_{i},y_{i}\right)$ denotes the *i*th most relational RP that correspond to the coordinates (*i* = 1, 2, …, *K*).

## 4. Experiments and Results

In this study, the experiments are conducted to evaluate the performance of the proposed fingerprint method. The evaluation metrics of the positioning performance are selected in Section 4.2. Several factors, such as K values that influence the positioning methods and selection of Max AP, are discussed in Section 4.3. The comparison of the positioning performance of the different methods under heterogeneous smartphones is provided in Section 4.4.

### 4.1. Experimental Setup

The positioning performance of the proposed algorithm is tested. The experiment is conducted in an office building of the School of Geodesy and Geomatics, Wuhan University. The office area measures 6.4 m × 12.8 m. The AP and RP locations are shown in Figure 5. Nine APs are distributed in the office as transmitters, and their locations are unknown. Two transmitter models are presented. The first model is a TL-WR842N wireless router, which has two transmitting antennas and only transmits 2.4 GHz band signals. The second model is a TL-WDR5620 wireless router, which can transmit two kinds of frequency band signals, namely, 2.4 GHz and 5 GHz. The TL-WDR5620 wireless router has three transmitting antennas. The green circles represent the RPs. The location of each RP is known. The blue symbol indicates the TL-WR842N wireless router, and another model is represented by the purple symbol. The distance between two consecutive RPs is 1.6 m. The red diamond symbols represent the TPs. A total of 28 RPs and 39 TPs are deployed in the office.

**Figure 6:**
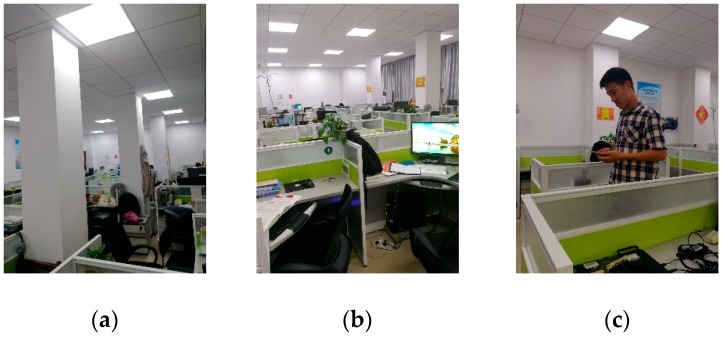
Photos of the experimental scene: (**a**,**b**) experimental scene photos from different angles and (**c**) data status collection.

On the software side, the RSS data are collected by a smartphone application developed by our research team. We perform the same setting in the process of collecting RSS data at each TP and RP. Four heterogeneous smartphones are used to collect the RSS data at a rate of 1 Hz. Table 1 shows the Wi-Fi modules from the four heterogeneous smartphones. Smartphones from distinct brands usually have different Wi-Fi modules from various manufacturers. In addition, various smartphone models from the same brand may have different Wi-Fi modules. The antenna positions of each smartphone may be different in smartphones of distinct brands. In order to eliminate the effect of body shadowing on positioning, the RSS data are collected from four different orientations: east, west, north, and south. The RSS data are collected in each direction for 15 s for a total of 60 s (60 RSS data) at each RP and TP. When RSS data are simultaneously collected from four heterogeneous smartphones at each TP and RP, each smartphone is in the hand, held at chest level, and maintains the same pose in the experimental scene of Figure 6. For convenience, an independent coordinate system is established in the office for positioning.

### 4.2. Evaluation Metrics of Positioning Performance

The mean absolute error (MAE), standard deviation (STD), and root mean square error (RMSE) are selected as accuracy evaluation metrics to evaluate positioning performance. The absolute errors are calculated by the Euclidean distances between the estimated and true coordinates at TPs. Uncertainty is commonly used in measurements [53]. Uncertainty of measurement refers to doubt on the result of any measurement. Error refers to the difference between the measured and true values. Uncertainty is a quantification of the doubt about the measurement result. Here, standard uncertainty (STU) is used as an evaluation metric of positioning performance. The definitions of the evaluation parameters are listed in Table 2.

### 4.3. Parameter Analysis of Different Positioning Methods

In the proposed method, some parameter settings will affect the positioning accuracy. The positioning results with different parameters, namely, the K value and number of Max AP, are analysed to determine the optimal parameters of the method for achieving the optimal positioning performance. We firstly rank all APs by using the received RSS data from each AP in a descending order at each RP to determine the number of Max AP. Subsequently, we select the Top-N APs from the descending sequence at each RP. Thereafter, the appearance frequency of each AP is counted in Top-N APs of all RPs. Finally, we select the *n* (n < N) most-counted APs.

**Figure 7:**
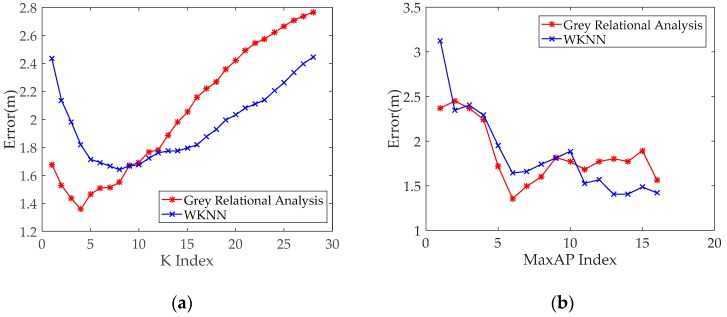
Positioning performance of the conventional fingerprint method and proposed method with the parameter changing: (**a**) parameter K and (**b**) the number of selected Max access points (APs).

Figure 7a shows that the mean errors of all TPs are calculated by the WKNN and proposed method and that the error changes with the K value. Figure 7a demonstrates that the positioning error shows a decreasing trend with the increase of K. The positioning error gradually increases when it is reduced to a certain extent. Accordingly, the positioning results of K = 4 and K = 8 are optimal with the GRA and WKNN algorithms in the office scenario, respectively.

Figure 7b presents that the mean errors of the WKNN algorithm and proposed GRA-based method changed with the increase in the selected Max APs. When the numbers of the selected Max APs are 6 and 13, the positioning results are optimal with the proposed GRA method and WKNN algorithm, respectively. However, when the numbers of the selected Max APs exceed 6 and 13, the positioning errors of the two methods demonstrate a slight change. This condition is attributed to all APs deployed in the same office, and RSS data from most APs have differences. When the number of selected Max APs is less than six, the positioning errors of the two methods have a notable change. This finding is attributed to the RSS fingerprint from a few APs at certain RPs, which are unstable due to existing pillars and office separators in the experimental environment. Thus, the optimal parameter setting can help improve the positioning accuracy.

### 4.4. Performance Evaluations of the Proposed Positioning Method

**Figure 8:**
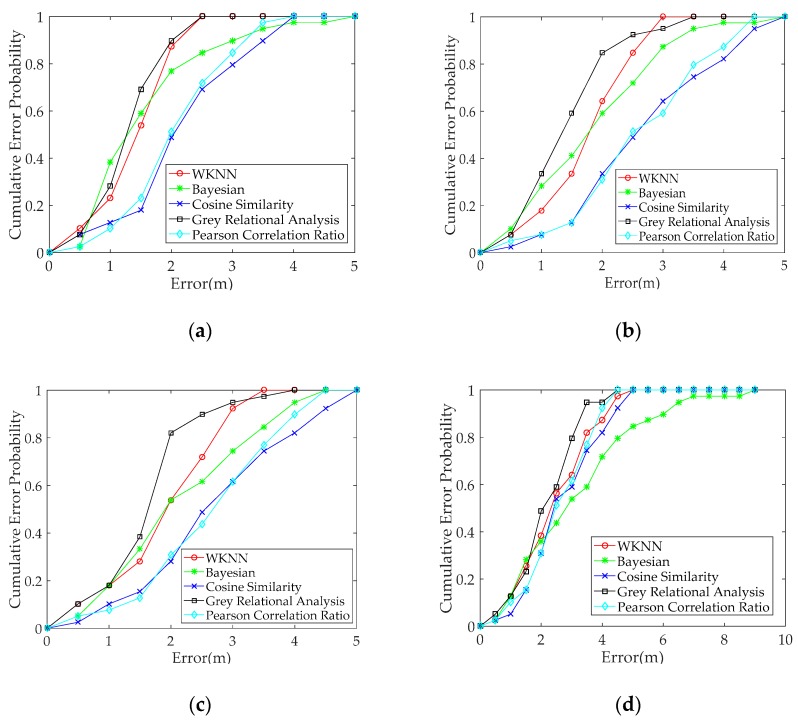
Comparison of positioning performance for different methods under heterogeneous and the same smartphones: Fingerprint data are collected with the Redmi 5plus smartphone in the offline phase, and the test data are collected with four different smartphones in the online phase: (**a**) Redmi 5plus, (**b**) Honor 9, (**c**) Redmi Note 3, and (**d**) Mi 6.

To test the positioning performance of the proposed GRA-based fingerprint method when heterogeneous smartphones are used, we selected RSS data from a pair of heterogeneous smartphones. The positioning performance of the different fingerprint methods are compared under heterogeneous and same smartphones. Our experimental results are shown in Figure 8 and Table 3. The RSS data from the same Redmi 5plus smartphone in the offline and online phases are used to verify the positioning performance of different methods when the same smartphones are used. The result is shown in Figure 8a and Table 3. The positioning error probability within 1.5 m for the proposed method based on GRA is better and above 10% compared with the other positioning methods based on Euclidean distance and similarity. The mean positioning error of the proposed fingerprint method is reduced by more than 0.1 m compared with Bayesian, cosine similarity (CS), and Pearson correlation ratio (PCR), and the proposed method enhances the positioning accuracy by more than 8% compared with the three other methods. The positioning performance of the proposed method slightly improved when the same smartphone is used.

In this work, we mainly consider the positioning performance when heterogeneous smartphones are used. Three pairs of heterogeneous smartphones are compared in Figure 8b–d and Table 3. The probability of error within 2 m for the proposed GRA-based method is better by 20% compared with the other positioning methods in Figure 8b,c and above 10% in Figure 8d. The mean positioning error of the proposed fingerprint method is reduced by 0.3 m, and the proposed method enhances the positioning accuracy by more than 15% compared with the four other methods in Table 3. The positioning performance of the proposed GRA-based fingerprint method is improved when heterogeneous smartphones are used. In addition, the positioning performances of CS and PCR are mostly unsatisfactory. This condition is attributed to the methods, which only utilise similarity to match fingerprint, and the fingerprint and test data do not effectively match. However, the fingerprint and test data can achieve optimal matching when the proposed method uses heterogeneous smartphones. In Table 3, the RMSE of the proposed fingerprint method is also reduced compared with the other methods. The positioning precision of the proposed fingerprint method is improved under heterogeneous smartphones. In Table 3, we can see that the STD and STU values of the proposed GRA method is minimal among the all methods, which indicates that the proposed method has good stability and availability in positioning performance. Table 3 also shows that the positioning errors of all methods that use heterogeneous smartphones are larger than those that use the same smartphone. The result indicates that the positioning performance of the proposed fingerprint method outperforms the other methods when heterogeneous smartphones are used. The comparison results in Figure 8b–d) shows that the positioning errors of all methods for any two pairs of different smartphones vary. By contrast, the positioning performance of the proposed fingerprint method is optimal in each pair of heterogeneous smartphones.

**Figure 9:**
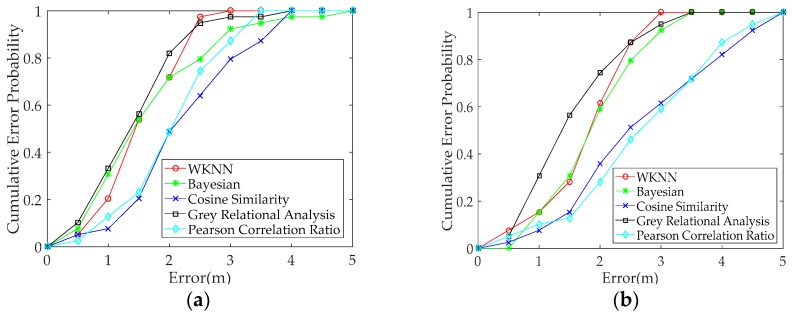

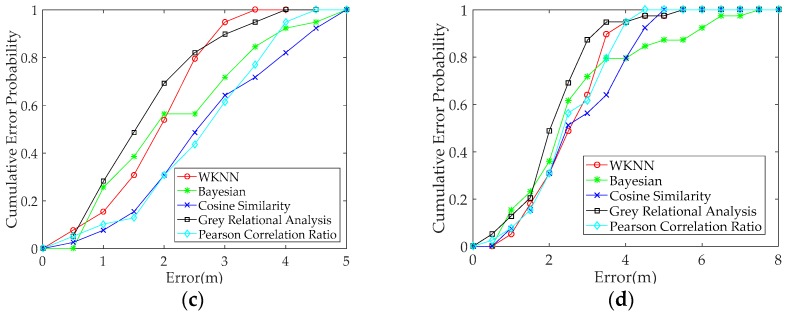
Comparison of positioning performance for different methods under heterogeneous and the same smartphones: Fingerprint data are collected with the Honor 9 smartphone in the offline phase, and the test data are collected with four heterogeneous smartphones in the online phase: (**a**) Honor 9, (**b**) Redmi 5plus, (**c**) Redmi Note 3, and (**d**) Mi 6.

To further verify the positioning performance of the proposed GRA-based fingerprint method, we selected RSS data from another pair of heterogeneous smartphones. The positioning performance of the different fingerprint methods are compared under heterogeneous and the same smartphones. The experimental results are shown in Figure 9 and Table 4. Here, the RSS data from the Honor 9 smartphone are used as fingerprint data and the RSS data of four different smartphones are used as test data. In Figure 9, the positioning performance of the proposed GRA-based fingerprint method is also improved under heterogeneous smartphones. Table 4 shows that the mean positioning error of the proposed fingerprint method is also reduced compared to the others. The positioning accuracy of the proposed fingerprint method outperforms other methods when heterogeneous smartphones are used.

In the comparative analysis of positioning performance using two pairs of heterogeneous smartphones, the positioning accuracies of the GRA-based fingerprint method are both improved compared with other positioning methods. The positioning performance of the proposed fingerprint method outperforms other methods when heterogeneous smartphones are used. These results indicate that the proposed fingerprint method has good stability to solve the heterogeneity problem. The positioning accuracies of all methods are unsatisfactory when the test data are from the Mi 6 smartphone and when the fingerprint data are from the Honor 9 or Redmi 5plus smartphones. The results suggest that the Wi-Fi module of the Mi 6 smartphone may exhibit certain problems in the collected RSS data.

The positioning performance of the proposed GRA-based method is compared with those based on RSS difference when the same and heterogeneous smartphones are used. The RSS data from Redmi 5plus are used as fingerprint data. By contrast, the RSS data from the Honor 9 and Redmi 5plus smartphones are used as the test data. Here, we illustrate the proposed method based on GRA and other existing methods, including DIFF, SSD, and HLF, based on RSS difference. The WKNN algorithm is selected to estimate positioning performance. The RSS method is added for comparison, which only uses raw RSS data. The positioning performance of the proposed method based on GRA is evaluated by comparing the positioning performance of various preprocessing methods based on RSS difference. In addition, the LCS method is compared with other methods, which uses only relative relationship information among RSS values.

**Figure 10:**
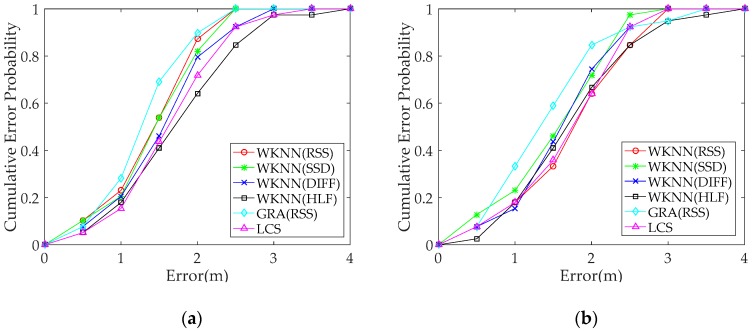
Comparison of positioning performance for different methods under heterogeneous and the same smartphones: The cumulative error probability represents the cumulative probability of absolute error of each TP. Fingerprint data are collected with the Redmi 5plus smartphone in the offline phase, and the test data are collected with different smartphones in the online phase: (**a**) Redmi 5plus and (**b**) Honor 9.

Figure 10 and Table 5 show that the mean positioning error of the proposed fingerprint method is reduced by more than 0.1 m when the same smartphone is used and above 0.15 m when heterogeneous smartphones are used (Table 4). The positioning performance of the proposed method based on GRA outperforms the other methods based on the RSS difference when heterogeneous smartphones are used. The positioning performance of the WKNN algorithms based on RSS difference is good when the heterogeneous smartphones are used in the WKNN algorithms. By contrast, the WKNN algorithm using raw RSS data has the better performance when the same smartphone is used. This outcome illustrates the adverse influence of the methods based on RSS difference on positioning accuracy. In Figure 10, the positioning performance of the DIFF method is slightly better than that of SSD. That is because the SSD method only preserves the difference between adjacent APs, resulting in loss of certain useful information, thereby affecting the overall performance. The positioning performance of the LCS method is not ideal when heterogeneous smartphones are used because the number of APs is insufficient in the experiment environment. Thus, the proposed GRA-based method is better compared with other methods.

## 5. Conclusions

RSS data from heterogeneous smartphones have differences even if the RSS data are measured from the same AP and location. Thus, the impacts of using heterogeneous smartphones on the fingerprint method are further analysed. We find that the positioning performance of conventional fingerprint algorithms are degraded. GRA theory is introduced to Wi-Fi indoor positioning to address this heterogeneous smartphones problem, and a device calibration-free fingerprint method based on GRA is proposed. In the proposed method, GRC are determined by the RSS absolute difference between the elements of the comparability sequence of each RP and the elements of the reference sequence of TP and GRD is determined by the GRC between reference and comparability sequences. The TP location is subsequently determined by the GRD information between TP and each RP. The proposed method can adapt to the changes in RSS data between two vectors better than other fingerprint methods in terms of similarity. The proposed method can also avoid missing the raw RSS fingerprint information compared with other RSS difference-based fingerprint methods. All experimental results show that the mean positioning error and RMSE of the proposed fingerprint method are reduced relative to other positioning methods when heterogeneous smartphones are used, which indicates that the positioning accuracy and precision of the proposed fingerprint method are improved. The experimental results also show that the STD and STU values of the proposed GRA method is minimal among the all methods in the heterogeneous smartphones, which indicates that the proposed method has good stability and availability. Accordingly, the proposed fingerprint method based on GRA has an optimal positioning performance when heterogeneous smartphones are used. However, the area of the experiment environment is limited. We will further conduct experiments to verify reliability of the proposed GRA method in other scenarios and within large experimental areas in the next step.

## Figures and Tables

**Table 1:** Wi-Fi module conditions of heterogeneous smartphones.

Smartphones	Wi-Fi Module	Standards	Antenna Position
Redmi 5plus	Qualcomm-QFE2101	IEEE 802.11 a/b/g/n	Both near top and bottom
Honor 9	Broadcom-BCM43455XKUBG	IEEE 802.11 a/b/g/n/ac	Near upper left
Redmi Note 3	MT6630QP	IEEE 802.11 a/b/g/n/ac	Near bottom of the phone
Mi 6	Qualcomm-WCN3990	IEEE 802.11 a/b/g/n/ac	Near the four corners

**Table 2:** Definition of evaluation parameters.

Evaluation Parameters	Definition
Estimated TP Location	*TL*
True TP Location	*TL_truth_*
Absolute Error	$err_{TL}=\left|TL-TL_{truth}\right|$
Mean Absolute Error	$\bar{err_{TL}}=\frac{1}{N}\sum_{k=1}^{N}\left(\left|TL^{k}-TL^{k}{}_{truth}\right|\right)$
Standard Deviation	$\sqrt{\frac{1}{N-1}\sum_{k=1}^{N}{\left(\left|err_{TL}{}^{k}-\bar{err_{TL}}\right|\right)}^{2}}$
Standard Uncertainty	$\sqrt{\frac{1}{\left(N-1\right)N}\sum_{k=1}^{N}{\left(\left|err_{TL}{}^{k}-\bar{err_{TL}}\right|\right)}^{2}}$
Root Mean Square Error	$\sqrt{\frac{1}{N}\sum_{k=1}^{N}{\left(TL^{k}-T{L^{k}}_{truth}\right)}^{2}}$

**Table 3:** Detailed positioning errors for different methods under heterogeneous and the same smartphones.

Fingerprint Data	Testing Data	Method	MAE (m)	RMSE (m)	STD (m)	STU (m)
Redmi5 plus	Redmi5 plus	WKNN	1.3824	1.4908	0.5653	0.0905
		Bayesian	1.5139	1.8072	0.9998	0.1601
		CS	2.1217	2.3199	0.9505	0.1522
		GRA	**1.2682**	**1.3687**	**0.5214**	**0.0835**
		PCR	2.0520	2.1991	0.8009	0.1282
Redmi5 plus	Honor 9	WKNN	1.7378	1.8728	0.7235	0.1159
		Bayesian	1.8328	2.1251	1.0897	0.1745
		CS	2.6342	2.8704	1.1550	0.1849
		GRA	**1.3302**	**1.5240**	**0.7074**	**0.1133**
		PCR	2.5834	2.8058	1.1090	0.1776
Redmi5 plus	Redmi Note 3	WKNN	1.8772	2.0375	0.8025	0.1285
		Bayesian	2.1427	2.4307	1.1627	0.1862
		CS	2.6682	2.9119	1.1815	0.1892
		GRA	**1.5288**	**1.7205**	**0.7997**	**0.1281**
		PCR	2.6188	2.8272	1.0794	0.1728
Redmi5 plus	Mi 6	WKNN	2.4767	2.7356	1.1769	0.1885
		Bayesian	3.1304	3.6815	1.9630	0.3143
		CS	2.6776	2.9156	1.1690	0.1872
		GRA	**2.1027**	**2.3112**	**0.9718**	**0.1556**
		PCR	2.5581	2.7549	1.0360	0.1659

**Table 4:** Positioning errors for different methods under heterogeneous and the same smartphones.

Fingerprint Data	Testing Data	Method	MAE (m)	RMSE (m)	STD (m)	STU (m)
Honor 9	Honor 9	WKNN	1.5234	1.6392	0.6131	0.0982
		Bayesian	1.6259	1.8977	0.9913	0.1587
		CS	2.1477	2.3336	0.9248	0.1481
		GRA	**1.3958**	**1.5852**	**0.5612**	**0.0899**
		PCR	1.9985	2.1450	0.7892	0.1264
Honor 9	Redmi5 plus	WKNN	1.7649	1.8874	0.6778	0.1085
		Bayesian	1.8667	2.0040	0.7385	0.1183
		CS	2.6538	2.9000	1.1846	0.1897
		GRA	**1.5232**	**1.7072**	**0.6509**	**0.1042**
		PCR	2.6402	2.8611	1.1166	0.1788
Honor 9	Redmi Note 3	WKNN	1.8500	1.9953	0.7572	0.1212
		Bayesian	2.1344	2.4489	1.2163	0.1948
		CS	2.6740	2.9300	1.2132	0.1943
		GRA	**1.6273**	**1.8510**	**0.6935**	**0.1110**
		PCR	2.5801	2.7815	1.0527	0.1686
Honor 9	Mi 6	WKNN	2.5087	2.6865	1.0737	0.1719
		Bayesian	2.7199	3.1779	1.6652	0.2666
		CS	2.7084	2.9596	1.2088	0.1936
		GRA	**2.0892**	**2.3252**	**1.0341**	**0.1656**
		PCR	2.5180	2.7088	1.1219	0.1796

**Table 5:** Detailed positioning errors for different methods under heterogeneous and the same smartphones.

Fingerprint Data	Testing Data	Method	MAE (m)	RMSE (m)	STD (m)	STU (m)
Redmi5 plus	Redmi5 plus	WKNN(RSS)	1.3824	1.4908	0.5653	0.0905
		WKNN(DIFF)	1.4286	1.5508	0.6113	0.0979
		WKNN(SSD)	1.5372	1.6606	0.6362	0.1019
		WKNN(HLF)	1.6554	1.8069	0.7336	0.1175
		GRA(RSS)	**1.2682**	**1.3687**	**0.5214**	**0.0835**
		LCS	1.5736	1.7201	0.7037	0.1127
Redmi5plus	Honor 9	WKNN(RSS)	1.7378	1.8728	0.7074	0.1133
		WKNN(DIFF)	1.4969	1.6217	0.6520	0.1044
		WKNN(SSD)	1.5975	1.7202	0.6464	0.1035
		WKNN(HLF)	1.7105	1.8556	0.7288	0.1167
		GRA(RSS)	**1.3302**	**1.5240**	**0.6235**	**0.0998**
		LCS	1.6581	1.7881	0.6738	0.1086
